# An fMRI study of cognitive remediation in drug-naïve subjects diagnosed with first episode schizophrenia

**DOI:** 10.1007/s00508-021-01910-2

**Published:** 2021-07-13

**Authors:** Julia Furtner, Veronika Schöpf, Andreas Erfurth, Gabriele Sachs

**Affiliations:** 1 Department of Biomedical Imaging and Image-guided Therapy, Medical University of Vienna, Vienna, Austria; 2 1st Department of Psychiatry and Psychotherapeutic Medicine, Klinik Hietzing, Vienna, Austria; 3 Department of Psychiatry and Psychotherapy, Medical University of Vienna, Waehringer Guertel 18–20, 1090 Vienna, Austria

**Keywords:** Randomized study, Working memory, Atypical antipsychotics, Activation clusters, Neuronal activity

## Abstract

**Objective:**

The purpose of our functional magnetic resonance imaging (fMRI) study was to examine brain activity using a “1-back” paradigm as working memory task in drug-naïve subjects with first episode schizophrenia before and after cognitive remediation training.

**Methods:**

In this study 15 drug-naïve first episode subjects who met DSM-IV criteria for schizophrenia were randomized to receive either atypical antipsychotics (AP, *n* = 8) or atypical antipsychotics in combination with cognitive remediation therapy (AP + CR, *n* = 7), 11 subjects had a follow-up fMRI examination after therapy (AP, *n* = 5; AP + CR, *n* = 6).

**Results:**

In 4 of the 6 AP + CR subjects the number of activation clusters increased, whereas in 4 out of the 5 AP subjects the number of clusters decreased (mean number of clusters: AP + CR = 5.53, SD 12.79, AP = −5.8, SD 6.9).

**Conclusion:**

In this randomized study the number of activation clusters during a working memory task increased after cognitive remediation training. Our data show that neurobiological effects of cognitive remediation can be identified in the very early course of schizophrenia.

## Introduction

In subjects with schizophrenia cognitive dysfunction is well documented, including impairment in attention, memory, executive function and social cognition [1, 2]. These impairments are associated with poor social functioning and reduction of activities of daily living [3]. Working memory is a core neuropsychological component and is widely used in cognitive processes, such as language comprehension and production, reasoning and problem solving [4]. Working memory deficits are well known in subjects with schizophrenia regardless of stimulus modality [5]. Therefore, working memory dysfunction has been proposed as a cognitive endophenotype of schizophrenia. Several studies have demonstrated that subjects with schizophrenia show reduced activation of brain regions involved in working memory during a *n*-back test [6–9].

To improve cognition in subjects with schizophrenia it was suggested to combine pharmacotherapy with neuropsychological training [10]. A meta-analysis showed the positive effect of cognitive remediation programs in schizophrenia [11]. As reviewed by Mothersill and Donohoe [12], 15 functional neuroimaging studies have reported increased neural activation following cognitive training, with increased left prefrontal activation being the most frequent observation.

Few studies have directly examined working memory after cognitive remediation using functional imaging. Subramaniam et al. [13] performed a functional magnetic resonance imaging (fMRI) study of verbal working memory using the letter *n*-back task before and after an intensive computerized training. Better working memory was supported by enhanced prefrontal signal efficiency, which predicted better long-term functional outcome. Li et al. [14] examined the remediation effect of working memory (WM) training in subjects with schizophrenia with prominent negative symptoms. Increased brain activations were observed in the right insula and the right frontal lobe (sub-gyral) after WM training. To our knowledge, no studies have yet been performed in drug-naïve first episode subjects with schizophrenia.

The objective of our fMRI study was to evaluate brain activity during a *n*-back task in drug-naïve first episode subjects with schizophrenia before and after a cognitive remediation training program that focused on everyday living activities.

## Methods

### Subjects

A total of 15 drug-naïve first episode subjects who met DSM-IV criteria for schizophrenia (DSM-IV SCID-P) [15] were included in this study. Not included in the study were subjects with relevant somatic and psychiatric comorbidities, in particular substance-related and addictive disorders. Further exclusion criteria were previous treatment with antipsychotics and discontinuation of therapy, additional use of tranquilizers, or a mini mental status examination < 24. In addition, common magnetic resonance imaging (MRI) exclusion criteria were applied (i.e. claustrophobia, metallic implants, pregnancy and other conditions that may interfere with the diagnostic examination).

The subjects were inpatients of the Department of Psychiatry and Psychotherapy, Medical University of Vienna. Subjects received atypical antipsychotics (AP; first dose flexible) after the first MRI examination. During the treatment period no changes of dosage or substance were performed.

The participants gave informed consent and the study was approved by the local ethics committee. The study was carried out in accordance with the Declaration of Helsinki.

After baseline fMRI examination subjects were randomly assigned into two different cohorts. The first cohort received drug therapy with AP (*n* = 8); the second cohort received AP in combination with cognitive remediation therapy (AP + CR; *n* = 7).

### Cognitive remediation training

A standardized computer-based cognitive training provided by the COGPACK software package version 6.06 [16] was used and 12 single sessions, each lasting 60 min, were conducted.

### Functional MRI examination

Functional MR images were acquired on a 1.5 T MR scanner (Philips 1.5T Gyroscan Intera, Philips Medical Systems, Best, The Netherlands) using sequence parameters as follows: TE = 50 ms, TR = 3616 ms, number of repetitions = 100, slices = 36, slice thickness = 4 mm, gap = 4 mm, matrix size = 96 × 78 mm2, flip angle = 90°. Slices were aligned parallel to the anterior and posterior commissure.

A block designed “1-back” paradigm was used as working memory task. The paradigm consisted of 5 active blocks as well as 5 rest blocks with a duration of 36 s each resulting in an overall acquisition time for one fMRI sequence of 6 min. During the active conditions, subjects saw consecutive letters and were obligated to press a button when a letter was identical to the one immediately preceding it. A complete scanning session included 2 runs of the fMRI paradigm and a T1-weighted sequence for anatomical co-registration.

Subjects were scanned in supine position, without the application of any kind of sedatives. During fMRI scanning session the instructions and stimuli of the paradigm were visually presented via a special MR compatible monitor mounted on the head coil. Button press for the task were recorded using an MR compatible system.

Subjects wore earphones for further briefings of the physician and for noise reduction during the scanning time. They were asked to avoid any movements, especially head movements. Additionally, padding was arranged around the subject’s head to minimize movements and ensure as much as possible that the subject’s head was positioned the same way across the whole scan session.

### fMRI data analysis

Neuroimaging data were preprocessed, using SPM12 implemented in MatlabR2014b, with motion correction, slice-time correction, spatial normalization using DARTEL algorithm [17] and spatial smoothing (8-mm Gaussian kernel). Two functional runs were conducted for each of the 2 time points (before and after therapy). Two preprocessed functional runs for each time point were submitted to a fixed effects analysis (FFX) using modelled regressors for the 1‑back block design conducted (36 s on/off). Activity maps are reported on FWE (family-wise error) *p* < 0.005 level.

### Statistical analysis

Due to the small sample size for each group, we could not perform randomized t‑testing. The results are presented in a statistically descriptive manner. We decided to compare the number of clusters on an individual level.

## Results

### Subjects

Of these 15 subjects 11 underwent a second follow-up fMRI examination after therapy (first cohort, *n* = 5; second cohort, *n* = 6). The mean duration between the MR scans was 30 days (range 14–49 days) and four subjects had to be excluded from the study because no second fMRI was performed. The mean age of the 11 subjects (7 males and 4 females) was 24.6 years (range 18–37 years). All subjects were right-handed.

### Comparison of fMRI data before and after therapy

**Fig. 1:**
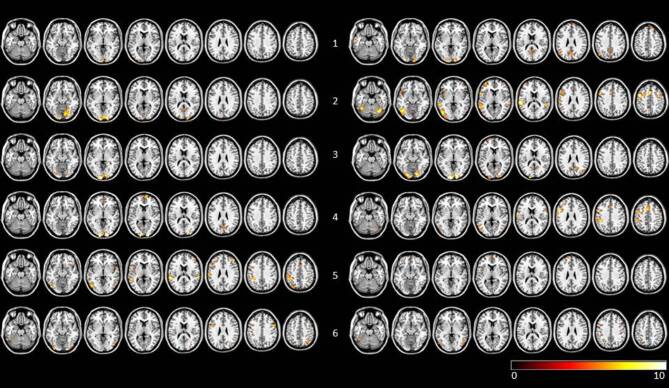
Visualization of FFX results of pretherapy and posttherapy *n*-back task runs for AP + CR subjects (FWE corrected, *p* < 0.005). Row labelling refers to subject number. To the left of the subject number individual scans before therapy are shown, to the right of the subject number the scans after therapy are shown. Data are visualized on a standard template using MRIcron. *AP* *+* *CR group* subjects with atypical antipsychotics and cognitive remediation, *FFX* fixed effects analysis, *FWE* family-wise error

**Fig. 2:**
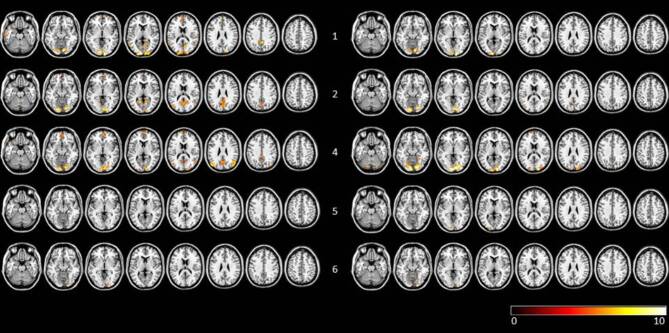
Visualization of FFX results of pre and post therapy *n*-back task runs for AP subjects (FWE corrected, *p* < 0.005). Row labelling refers to subject number. To the left of the subject number individual scans before therapy are shown, to the right of the subject number the scans after therapy are shown. Data are visualized on a standard template using MRIcron. *AP group* subjects with atypical antipsychotics, *FFX* fixed effects analysis, *FWE* family-wise error

In 4 of the 6 AP + CR subjects the number of clusters increased, whereas in 4 out of the 5 AP subjects the number of clusters decreased (mean number of clusters: AP + CR = 5.53, SD 12.79, AP = −5.8, SD 6.9; see Table 1 for details). Individual activation maps for AP + CR subjects before and after therapy are shown in Fig. 1 and for AP subjects in Fig. 2.

**Table 1:** Comparison of number of activation clusters for AP + CR and AP subjects before and after therapy. Calculations are based on FWE *p* < 0.005 corrected maps using fixed effects analysis

		Pre	Post	Difference
AP + CR	1	5	18	13
	2	13	28	15
	3	3	17	14
	4	16	31	15
	5	30	11	−19
	6	23	20	−3
AP	1	29	15	−14
	2	20	15	−5
	4	23	10	−13
	5	3	2	−1
	6	4	8	4

*AP* *+* *CR group* subjects with atypical antipsychotics and cognitive remediation, *AP group* subjects with atypical antipsychotics, *FWE* Family-Wise Error

## Discussion

To our knowledge, this is the first study in drug-naïve subjects with first episode schizophrenia using functional MR imaging to identify correlates of working memory-related hemodynamic responses. In our study, the number of activation clusters during a working memory task increased after COGPACK training. Our data show that neurobiological effects of cognitive remediation can be identified in the very early course of schizophrenia.

The course of cognitive deficits has been described to be different between early and late stages of schizophrenia [18, 19]. Early detection of specific symptoms in schizophrenia [20–23] seems to be crucial and may also have a prognostic value [24]; early intervention in schizophrenia spectrum disorders appears to be important [25–28] and it has been suggested that cognitive training should be provided as early as possible in the prodromal phases of schizophrenia in order to use the full rehabilitative potential of the subjects [29]. Indeed, Bowie et al. [30] found that cognitive remediation in the early course leads to greater cognitive improvement than in later stages of schizophrenia. In addition, Ventura et al. [31] found that in first-episode schizophrenia cognitive remediation can also improve negative symptoms and has an impact on social functioning. A longitudinal functional brain imaging study in the early course of schizophrenia showed enhanced activation in the right dorsolateral prefrontal cortex after cognitive enhancement therapy during 2 years of treatment [32]. It has been suggested that these changes are accompanied by a functional reorganization of neural networks and that cognitive remediation might have a neuroprotective effect [33, 34].

Although our data are limited and preliminary, and special caution is needed in their interpretation, they support the hypothesis that early in the course of schizophrenia, cognitive remediation results in cognitive improvement that is associated with increasing neuronal activity.

### Key points

The combination of a cognitive remediation program with atypical antipsychotics was accompanied by neurobiological changes in drug-naïve subjects with first episode schizophrenia.

The number of fMRI activation clusters during a working memory task increased after COGPACK training.

## References

[1] Sachs G, Steger-Wuchse D, Kryspin-Exner I, Gur RC, Katschnig H (2004). Facial recognition deficits and cognition in schizophrenia. Schizophr Res.

[2] Keefe RS, Harvey PD (2012). Cognitive impairment in schizophrenia. Handb Exp Pharmacol.

[3] Green MF (2016). Impact of cognition and social impairment on functional outcomes in patients with schizophrenia. J Clin Psychiatry.

[4] Logie RH, Cowan N (2015). Perspectives on working memory: introduction to the special issue. Mem Cogn.

[5] Lee J, Park S (2005). Working memory impairments in schizophrenia: a meta-analysis. J Abnorm Psychol.

[6] Ettinger U, Williams SC, Fannon D, Premkumar P, Kuipers E, Möller HJ (2011). Functional magnetic resonance imaging of a parametric working memory task in schizophrenia: relationship with performance and effects of antipsychotic treatment. Psychopharmacology.

[7] Marenco S, Stein JL, Savostyanova AA, Sambataro F, Tan HY, Goldman AL (2012). Investigation of anatomical thalamo-cortical connectivity and FMRI activation in schizophrenia. Neuropsychopharmacology.

[8] Jiang S, Yan H, Chen Q, Tian L, Lu T, Tan HY (2015). Cerebral inefficient activation in schizophrenia patients and their unaffected parents during the n-back working memory task: a family fMRI study. PLoS ONE.

[9] Li X, Yi ZH, Lv QY, Chu MY, Hu HX, Wang JH (2019). Clinical utility of the dual n-back task in schizophrenia: a functional imaging approach. Psychiatry Res Neuroimaging.

[10] Marder SR (2006). Drug initiatives to improve cognitive function. J Clin Psychiatry.

[11] Wykes T, Huddy V, Cellard C, McGurk SR, Czobor P (2011). A meta-analysis of cognitive remediation for schizophrenia: methodology and effect sizes. Am J Psychiatry.

[12] Mothersill D, Donohoe G (2019). Neural effects of cognitive training in schizophrenia: a systematic review and activation likelihood estimation meta-analysis. Biol Psychiatry Cogn Neurosci Neuroimaging.

[13] Subramaniam K, Luks TL, Garrett C, Chung C, Fisher M, Nagarajan S (2014). Intensive cognitive training in schizophrenia enhances working memory and associated prefrontal cortical efficiency in a manner that drives long-term functional gains. Neuroimage.

[14] Li X, Chu MY, Lv QY, Hu HX, Li Z, Yi ZH (2019). The remediation effects of working memory training in schizophrenia patients with prominent negative symptoms. Cogn Neuropsychiatry.

[15] First MG, Spitzer RL, Gibbon M, Williams JB (1995). Structured clinical interview for DSM-IV patient edition (SCID-P).

[16] Marker KR. COGPACK. The Cognitive Training Package Manual. Heidelberg & Ladenburg: marker software; 2002.

[17] Ashburner J (2007). A fast diffeomorphic image registration algorithm. Neuroimage.

[18] Matsuda Y, Sato S, Hatsuse N, Watanabe Y, Kishimoto T, Ikebuchi E (2014). Neurocognitive functioning in patients with first-episode schizophrenia 1 year from onset in comparison with patients 5 years from onset. Int J Psychiatry Clin Pract.

[19] Kim SJ, Shim JC, Kong BG, Kang JW, Moon JJ, Jeon DW (2015). Differences in cognitive function and daily living skills between early- and late-stage schizophrenia. Int J Psychiatry Clin Pract.

[20] Sachs G, Winklbaur B, Jagsch R, Keefe RS (2011). Validation of the German version of the brief assessment of cognition in schizophrenia (BACS)—preliminary results. Eur Psychiatry.

[21] Sachs G, Lasser I, Purdon SE, Erfurth A (2021). Screening for cognitive impairment in schizophrenia: psychometric properties of the German version of the Screen for Cognitive Impairment in Psychiatry (SCIP-G). Schizophr Res Cogn.

[22] Mucci A, Vignapiano A, Bitter I, Austin SF, Delouche C, Dollfus S (2019). A large European, multicenter, multinational validation study of the Brief Negative Symptom Scale. Eur Neuropsychopharmacol.

[23] Berendsen S, Kapitein P, Schirmbeck F, van Tricht MJ, McGuire P, Morgan C (2020). Pre-training inter-rater reliability of clinical instruments in an international psychosis research project. Schizophr Res.

[24] Tognin S, Catalan A, Modinos G, Kempton MJ, Bilbao A, Nelson B (2020). Emotion recognition and adverse childhood experiences in individuals at clinical high risk of psychosis. Schizophr Bull.

[25] Penttilä M, Jääskeläinen E, Hirvonen N, Isohanni M, Miettunen J (2014). Duration of untreated psychosis as predictor of long-term outcome in schizophrenia: systematic review and meta-analysis. Br J Psychiatry.

[26] Goff DC, Falkai P, Fleischhacker WW, Girgis RR, Kahn RM, Uchida H (2017). The long-term effects of antipsychotic medication on clinical course in schizophrenia. Am J Psychiatry.

[27] Goff DC, Li C, Thorpe L (2020). Does early intervention improve the long-term course of schizophrenia?. Am J Psychiatry.

[28] Tognin S, van Hell HH, Merritt K, Winter-van Rossum I, Bossong MG, Kempton MJ (2020). Towards precision medicine in psychosis: benefits and challenges of multimodal multicenter studies-PSYSCAN: translating neuroimaging findings from research into clinical practice. Schizophr Bull.

[29] Rauchensteiner S, Kawohl W, Ozgurdal S, Littmann E, Gudlowski Y, Witthaus H (2011). Test-performance after cognitive training in persons at risk mental state of schizophrenia and patients with schizophrenia. Psychiatry Res.

[30] Bowie CR, Grossman M, Gupta M, Oyewumi LK, Harvey PD (2014). Cognitive remediation in schizophrenia: efficacy and effectiveness in patients with early versus long-term course of illness. Early Interv Psychiatry.

[31] Ventura J, Subotnik KL, Gretchen-Doorly D, Casaus L, Boucher M, Medalia A (2017). Cognitive remediation can improve negative symptoms and social functioning in first-episode schizophrenia: a randomized controlled trial. Schizophr Res.

[32] Keshavan MS, Eack SM, Prasad KM, Haller CS, Raymond Y, Cho RY (2017). Longitudinal functional brain imaging study in early course schizophrenia before and after cognitive enhancement therapy. Neuroimage.

[33] Penadés R, González-Rodríguez A, Catalán R, Segura B, Bernardo M, Junqué C (2017). Neuroimaging studies of cognitive remediation in schizophrenia: a systematic and critical review. World J Psychiatry.

[34] Penadés R, Franck N, González-Vallespí L, Dekerle M (2019). Neuroimaging studies of cognitive function in schizophrenia. Adv Exp Med Biol.

